# Targeting Difficult Protein-Protein Interactions with Plain and General Computational Approaches

**DOI:** 10.3390/molecules23092256

**Published:** 2018-09-04

**Authors:** Mariarosaria Ferraro, Giorgio Colombo

**Affiliations:** 1 Istituto di Chimica del Riconoscimento Molecolare, CNR, Via Mario Bianco 9, 20131 Milano, Italy; mariar.ferraro@gmail.com; 2 Dipartimento di Chimica, Università di Pavia, V.le Taramelli 10, 27100 Pavia, Italy

**Keywords:** molecular dynamics, proteins, molecular recognition, protein protein interactions

## Abstract

Investigating protein-protein interactions (PPIs) holds great potential for therapeutic applications, since they mediate intricate cell signaling networks in physiological and disease states. However, their complex and multifaceted nature poses a major challenge for biochemistry and medicinal chemistry, thereby limiting the druggability of biological partners participating in PPIs. Molecular Dynamics (MD) provides a solid framework to study the reciprocal shaping of proteins’ interacting surfaces. Here, we review successful applications of MD-based methods developed in our group to predict interfacial areas involved in PPIs of pharmaceutical interest. We report two interesting examples of how structural, dynamic and energetic information can be combined into efficient strategies which, complemented by experiments, can lead to the design of new small molecules with promising activities against cancer and infections. Our advances in targeting key PPIs in angiogenic pathways and antigen-antibody recognition events will be discussed for their role in drug discovery and chemical biology.

## 1. Introduction

The existence of complex wirings in protein-protein interaction (PPI) networks finely modulates the inner working of the circuits at the basis of cell life. Their correct or incorrect regulation is naturally linked to the evolution of cells towards normal or diseases states. Being so important in disparate aspects of cellular functions, it comes as no surprise that PPIs have been the subject of intense studies over the last few years [1,2,3,4,5,6]. Understanding protein-protein recognition and binding entails shedding light on the regulatory mechanisms, as well as deepening our knowledge of the relationships between protein sequences, structure and their interactions [7]. From the practical point of view, our ability to master PPIs could play a key role in the fields of medicinal chemistry, chemical and synthetic biology. Indeed, not only could there be room for new strategies aimed at rewiring signaling pathways for synthetic biology, but also to develop new molecules against complex or yet undrugged targets, for diagnostic and therapeutic purposes [2].

In general, PPIs represent a class of interactions of high complexity. Structural and biophysical studies have shown that the features of the regions involved in interactions with other partners are diverse and multifaceted: contact surfaces may be large compared to the ones involved in protein-small molecule interactions; they are often flat and lack the grooves and crevices which are engaged by small molecules, and finally, they can be highly dynamic to favor adaptation to alternative binding partners [1,8]. Nonetheless, several methods and strategies to discover orthosteric, adaptive and allosteric inhibitors, as well as those pointing at PPIs promoters and stabilizers have been developed and excellently reviewed by Cesa and coworkers [9,10].

From the experimental point of view, mutational studies have shown that limited subsets of interface residues actually contribute to the affinity between the binding partners. In the context of targeting interface plasticity, flexible peptides selected by high-throughput screening (HTS) methods (such as phage display or large library screenings) have shown the ability to outcompete the natural partner by adapting to the interaction surface [2,11]. Similarly, HTS of small molecules against biochemically-reconstituted complexes have led to the identification of useful compounds with phenotypic effects when tested in cells. However, in this case, instead of directly monitoring physical interaction, researchers set out to characterize the functional consequences of the inhibition of a particular class of PPIs as a surrogate for binding measurements [9,12]. This is an interesting example of application of HTS methods to find modulators of PPI networks that highlights the importance of considering with care approaches to target challenging PPIs, like those intrinsically characterized by weak or transient interactions and for which classical HTS-based detection is not suitable [9,10].

These facts vividly portray a situation in which many aspects of protein-protein interactions have been investigated with success. Despite this sophistication and advancement, there is still no experimental technique that can predict at atomic level the determinants of what makes a protein surface an interacting one, or defines rules for the design of new molecular entities with applications in chemical biology or drug development. To tackle these problems, we have little choice but to turn to theoretical and computational approaches.

Theoretical methods to predict interacting surfaces of a protein of known structure fall into three main classes: (a) statistical approaches, (b) structural techniques, and (c) molecular dynamics (MD)-based methods. Statistical approaches relate an amino acid sequence to known 3-D structures and known tendencies for specific sequence motifs to be localized within interaction areas. Nowadays, these methods are widely used also in combination with coevolution concepts [13]. However, they provide no information regarding possible alternative conformations. Structural techniques use information on the geometric patterns of backbones and side chains involved in PPIs to recognize whether they are present in previously uncharacterized instances [14]. However, these methods cannot be used to describe the dynamics underlying the recognition process.

MD simulations represent a prime tool to characterize both the networks of interactions and the range of alternative states that can determine whether a protein surface may actually be an interacting one, and/or the dynamics of the processes of molecular recognition with binding partners [15,16,17,18,19]. In some cases, MD simulations can be integrated with quantum calculations to describe complex reactive processes at the basis of downstream recognition events [20]. In this focused perspective, we will discuss cases from our own experience where MD-based approaches have been used to derive compact physico-chemical descriptors of peptide-protein interactions that could be efficaciously translated into the discovery of new active small molecules, and to predict specific types of protein-protein interaction interfaces (namely those involved in antigen-antibody binding). In general, our framework entails the use of computational results for the design and experimental tests of active chemical tools to probe a certain PPI. Such chemical probes, indeed, represent the direct products of our ability to understand and suitably mimic the determinants of an interaction: in this view, they are designed to target and perturb a specific area and to report on the effects of such perturbation in cells. At the end of this paper, we will discuss possible perspectives in the development of novel therapeutics, such as drugs with novel mechanisms and synthetic antigens for vaccination.

## 2. MD-Based Methods for Studying PPIs: Studying Peptides to Develop Novel Small-Molecule Anticancer Drug Candidates

The availability of a general framework to design molecules that meet the specific structural/dynamical requirements to perturb a certain function is both a necessity and an opportunity towards innovative discovery of therapeutics and chemical tools. A full understanding of the roles of different sub-states of a molecular interacting system will allow a more rational design of the chemical probes we need to target a specific PPI; this can potentially translate into our ability to control the responses obtained by any system in which the interaction is involved.

**Figure 1:**
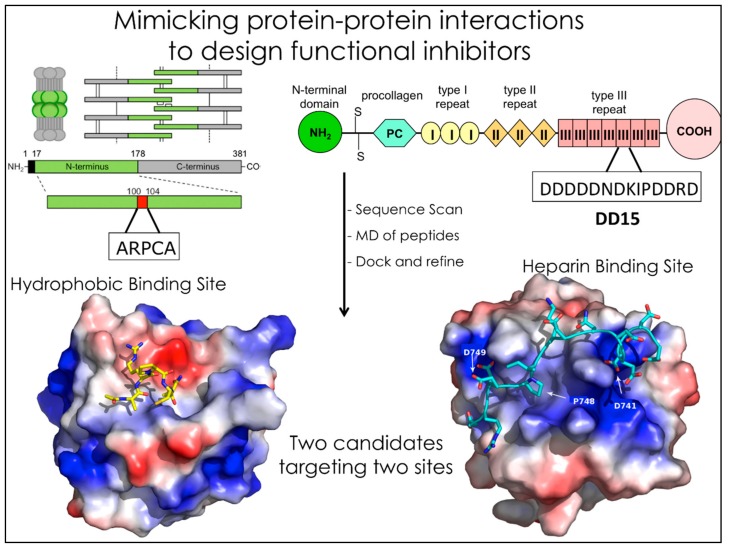
Simplified scheme depicting the identification of specific binding sequences in large multidomain proteins. Here, the cases of TSP1 and PTX3 binding to FGF2 are shown.

Building on these considerations, we built a pipeline for the design of small molecules mimics of peptides known to interrupt relevant PPIs in the control of angiogenesis, the process of vascular growth widely exploited by tumors to support their own development and diffusion. To proceed along these lines, we started from the experimentally characterized interactions between the protein Fibroblast Growth Factor-2 (FGF2) and peptides derived from two large extracellular multi-domain proteins known to interact with it, namely Thrombospondin-1 (TSP1) and Pentraxin-3 (PTX-3) [21,22,23,24,25,26]. TSP1 and PTX3 are two distinct endogenous inhibitors of FGF2, which engage the target with different mechanisms at different interfaces [25,26] (Figure 1).

Although both proteins inhibit FGF-dependent angiogenic responses, in mechanisms related to tumor onset and development such inhibitory activity is not present and FGF2 is free to engage tyrosine kinase (TK) FGF Receptors (FGFR1-4). In presence of heparan sulphate proteoglycans (HSPGs), FGF2 binds the TKR subtypes to form HSPG/FGF/FGFR ternary complexes [27]. Activation of the FGF/FGFR system is implicated in key steps of tumor growth and progression [27]. Furthermore, compensatory up-regulation of the FGF/FGFR system may facilitate the escape from endothelial growth factor (VEGF) blockade [27]. Thus, the development of anti-FGF/FGFR targeting agents represents an urgent medical need in cancer therapy.

In this context, we started by examining the possibility of exploiting the dynamic cross-talk between FGF2 and a binding peptide in drug-candidate selection [24,28]. Our reasoning was based on the idea that molecular recognition entails a two-way influence between the interacting partners, whereby FGF2 flexibility determines the peptide conformation while the peptide poses dictate the stereochemical organization of the binding site. This dynamic adaptation is used to define the principal pharmacophoric determinants responsible for forming a stable complex. To dissect the sequence determinants of the interaction between TSP1 and FGF2, we first analyzed the binding profile of an array of peptides from a library of TSP1-derived synthetic compounds. The peptide array was designed based on the sequence of the type III repeats: 237 20-mer peptides with partially overlapping sequences (19-amino acid overlaps) were synthesized and covalently linked to polypropylene cards. The binding of biotinylated FGF2 (10 μg/mL) to the peptides was then tested. Bound FGF-2 was detected with peroxidase-conjugated streptavidin and the peroxidase substrate 2,2′-azino-di-3-ethylbenzthiazoline sulfonate (ABTS). Color development was quantified with a CCD camera, which reported on the affinities of different sequences for the target FGF2 [24].

Upon focusing on the best binding sequences, SPR identified peptide DDDDDNDKIPDDRDN, labeled DD15, as the one with the highest affinity. Sensorgrams indicated a dose-dependent binding of DD15 to FGF-2, with an association rate *K*_on_ of 19.7 ± 2.0 M^−1^·s^−1^ and a dissociation rate *K*_off_ of (5.5 ± 0.8) × 10^−4^ s^−1^, with a resulting *K_d_* of 28.0 μM. The peptide was located in the type III repeats of TSP1 [24,28] (Figure 1).

MD simulations were extensively performed on DD15 to obtain a pool of conformations, which were grouped into clusters. Simulations for DD15 were started from a fully extended conformation of the peptide to eliminate possible conformational biases. An initial representative conformation for the peptide was obtained by conformational search using the Systematic Unbounded Multiple Minimum (SUMM) method with the AMBER force field and the Polak-Ribiere Conjugate Gradient (PRCG) minimization method [29]. The minimum conformation obtained from this preliminary calculation was then subjected to MD refinement in explicit water solvent. The resulting trajectories were analyzed by the structural clustering method described by Daura et al. [30]. The most representative structures of DD15 obtained after cluster analysis of the trajectory were subjected to multiple docking runs on the surface of FGF-2 (PDB code 1fq9) using the program AUTODOCK, as described in [31]. The representative structure of the most populated cluster obtained from the docking runs, corresponding also to the free energy minimum, was used for successive MD refinement, which was carried out at 300K in explicit SPC water using the GROMACS software. This step was aimed essentially to characterize ligand-receptor reciprocal adaptation at atomic level.

**Figure 2:**
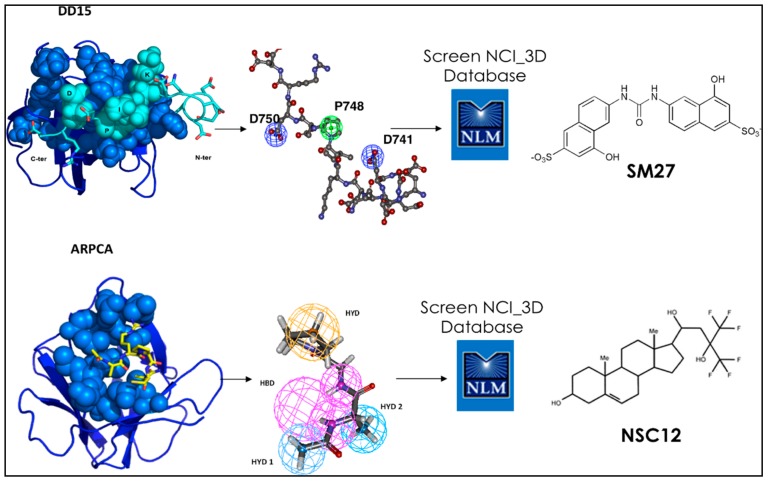
Definition of the most relevant contacts underlying the interaction between TSP1 and PTX3 derived peptides and FGF2, and their translation into pharmacophores for drug screening. Active small molecules sm27 and NSC12 are depicted.

Statistical analyses of the trajectories were next used to identify the stereochemical requirements the peptide must satisfy to ensure a stable binding to FGF. This information was translated into a pharmacophore model used to screen the NCI2003 small molecule databases. Briefly, the model was created using the central structure of the most populated cluster for the DD15·FGF-2 complex as a template on which to cast the design. The relative distances, orientations (dihedral angles) among the different groups of DD15, and the contacts (hydrophilic/hydrophobic) associated to the most persistent interactions with FGF-2 were retained as pharmacophoric determinants. The details of the procedure can be found in [24]. The screening of the NCI repository eventually led to the identification of three FGF-2-binding small molecules (Figure 2).

The lead compounds inhibited the angiogenic activity of FGF-2 in vitro, and in the Chick Chorioallantoic Membrane (CAM) assay, in vivo. Importantly, the discovered leads showed inhibiting properties comparable to the ones of the full length TSP-1 protein domain, which they were discovered from, at the same time featuring drug-like properties.

These results demonstrate the feasibility of integrating structure and dynamics to develop small molecule mimics of endogenous proteins as therapeutic agents [24,28]. It is important to underline here that MD revealed that both the small molecule and the peptide were able to engage the FGF2 interface involved in binding FGFR and heparin. Competition experiments further supported this finding.

This work was one of the first instances in which simulations and experiments were combined to target a difficult PPI. The surface on FGF2 is indeed large, flat and highly charged, all factors that together conspire against the possibility to define a druggable surface. In subsequent developments, the most potent compound, sm27, was used as a template for a similarity-based screening of small molecule libraries, followed by docking calculations and experimental studies. This allowed selecting seven binaphthalenic compounds that bound FGF2, inhibiting its binding to both heparin sulfate proteoglycans and FGFR. The compounds suppressed FGF2 activity in ex vivo and in vitro models of angiogenesis, with improved potency over sm27. Comparative analysis of the selected hits, complemented by NMR and biochemical analysis of four newly synthesized phenylamino-substituted naphthalene derivatives, allowed identifying the minimal stereochemical requirements to improve the design of naphthalene sulfonates as FGF2 inhibitors [32,33,34,35].

Next, we studied the interaction of a peptidic lead derived from the soluble pattern recognition receptor long-pentraxin 3 (PTX3) (Figure 1 and Figure 2). Human PTX3 overexpression inhibits tumor growth, angiogenesis and metastasis in heterotopic, orthotopic and autochthonous FGF-dependent tumor models by trapping FGF2 [36]. The acetylated pentapeptide Ac-ARPCA-NH_2_ (in single letter code, hereafter referred to as ARPCA), corresponding to the *N*-terminal amino acid sequence of PTX3 (100–104), was shown to act as a minimal anti-angiogenic FGF-binding peptide able to interfere with the formation of FGF/FGFR complexes [37]. We started from these observations to characterize ARPCA in solution and dock its principal conformations to FGF2. ARPCA was predicted to bind to a different region than DD15. Indeed, experimentally, it was unable to antagonize HSPGs.

Pharmacophore modeling of the interaction of ARPCA with FGF2 was next used for the identification of the first small molecule chemical (NSC12), which was shown to act as an orally active extracellular FGF trap with significant implications in cancer therapy. Indeed, in FGF-dependent murine and human tumor models, parenteral and oral delivery of NSC12 inhibits FGFR activation, tumor growth, angiogenesis and metastasis [36] (Figure 2).

Importantly, the characterization of a PPI by means of a minimal peptide led to the rational design of NSC12, which represents the first orally active small molecule ligand that can selectively prevent FGF2 from binding to FGFR and has interesting potential for anticancer drug development. 

Most interestingly, the two small FGF2-targeted molecules were predicted by computational approaches to bind different regions of FGF2. This fact was verified experimentally by competition experiments and NMR analyses [32,33,34,35].

These results strongly support the validity of computational approaches to investigate hard-to-drug PPIs, showing the ability to recapitulate the determinants of the binding process involving large multi-domain proteins (TSP1 and PTX3) and their endogenous target, for drug design applications. Furthermore, the diversity of the generated chemotypes and their ability to target different interaction surfaces open up attracting perspectives for drug development and drug-combination strategies. 

## 3. MD-Based Methods for Studying PPIs: The Case of Antibody-Antigen Interactions

As hard as they are to drug, protein-protein interaction regions offer, nonetheless, fresh opportunities for the discovery of molecules with therapeutic perspectives. This consideration may be particularly valid in the context of the development of strategies to tackle emerging pathogens or drug-resistant ones. Indeed, the spread of drug-resistance in pathogenic bacteria or the appearance of new viruses (Ebola, novel forms of aggressive influenza, Zika and dengue …) have severely limited the therapeutic efficacy of routinely used antibiotics, posing one of the most serious threats in modern medicine. In most of these cases, rapid diagnosis and vaccination represent the best option for the treatment of emerging infectious diseases. In fact, rapid and effective diagnosis can help preventing the spread of these threats in an increasing part of the population, while directing patients towards the best therapeutic options. In the last few years, it has become increasingly clear that, in order to develop biomolecules with both diagnostic and vaccine application potential, it is crucial to identify antigens on the surface of bacteria that are capable of eliciting a strong immune response, which is usually achieved through the production of (bactericidal) antibodies (Abs). In terms of diagnostic applications, the ability of antigens to proficiently interact with Abs can be exploited to develop probes that can reveal circulating antibodies produced in response to a specific infection in patient serum, blood or plasma samples. In terms of vaccine development, reactive protein antigens can be exploited in formulations aimed to elicit protective responses against successive pathogenic challenges. Even if vaccines have traditionally suffered from slow routine studies, sometimes providing viable products well after the peak in the epidemics, the advent of ‘Reverse Vaccinology’ (RV) has revolutionized the field, introducing a whole new strategy of antigen selection [38,39,40,41]. Starting from the full genome analysis of a pathogen or from the analysis of multiple pathogens of a certain family, RV antigen candidates that show key properties required for vaccine development (e.g., cell-surface exposure, ability to interact with/elicit Abs, protein stability, possibility to produce the protein antigens in recombinant form) are selected. To achieve such selection, RV makes use of complementary and synergistic methods, such as functional genomics, protein microarrays, and bioinformatics/computational biology. The reach of RV can be dramatically extended by the exploitation of atomic-level 3D information to engineer new biomolecules with improved immunoreactivity and/or biochemical properties [42,43,44,45]. 

**Figure 3:**
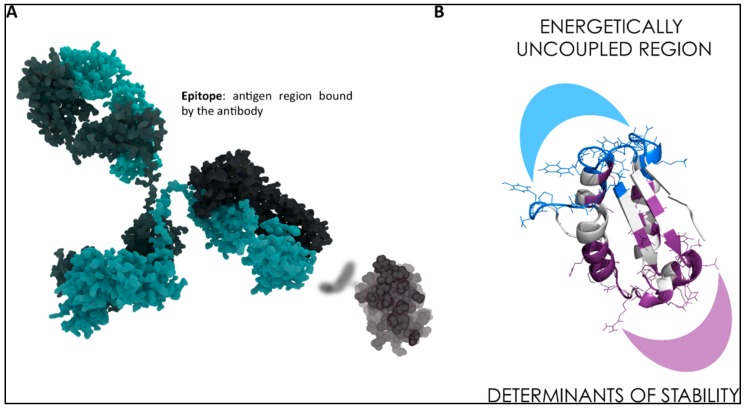
(**A**) Identification of the epitope region of an antigen binding to an antibody. (**B**) Simplified representation of the separation between stability and recognition regions in one protein antigen.

**Figure 4:**
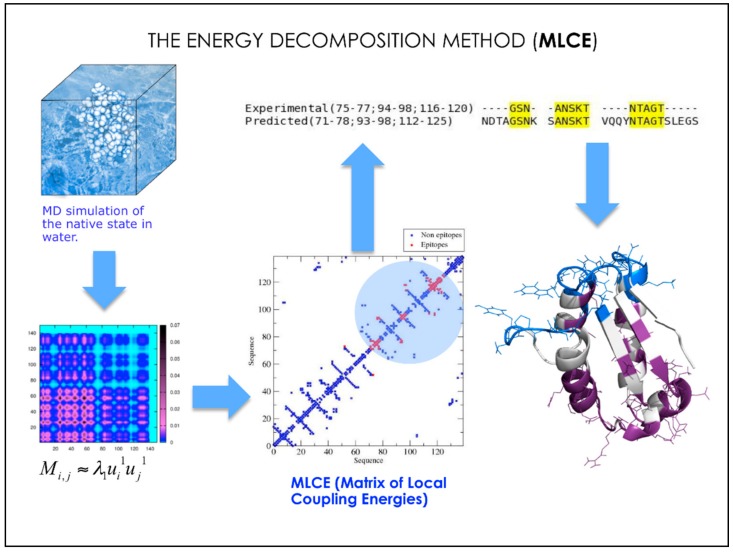
Schematics of how the MLCE algorithm works.

In chemical and physico-chemical terms, this comes down to identifying which regions in a protein antigen are the ones most likely to be immunoreactive with Abs. Such regions are called epitopes. In other words, one should detect the parts of the antigen that have the highest tendency to bind Abs. In this context, the problem is a particular case-study of protein-protein interactions (Figure 3). To meet this challenge, we have developed a simple computational strategy that aims at predicting Abs-binding epitopes starting only from the consideration of the structure, interactions and conformational dynamics of the antigen [46,47] (Figure 4).

Our approach starts from the idea that recognition sites may correspond to localized regions on the surface with low-intensity energetic couplings with the folding core of the protein which antigen belongs to: such minimal coupling to the rest of the structure can in principle allow the regions to sustain the conformational changes necessary to adapt to a binding partner. Indeed, in many cases, PPI regions have been shown to be endowed with flexibility features. 

We thus set out to identify non-optimized, low-intensity energetic interaction networks in the protein structure isolated in solution and then to benchmark the results against antibody complexes. Interestingly, it was found that the method could successfully identify binding sites located on the protein surface that are accessible to putative binding partners.

To identify localized surface regions with non-optimized interactions, we combined the analysis of internal protein energetics with the topological structural information obtainable from the contact matrix of either the crystal structure of the protein or the representative structure extracted from the MD trajectory (Figure 4).

The analysis of energetics derives from the energy decomposition method (EDM) [48,49,50,51,52,53,54,55]: specifically, the method provides a simplified view of residue-residue pair interactions, extracting the strongest and weakest residue pair-interactions and their contributions to energetic stability of a certain 3D structure.

In the case of a protein of length N, the *N* × *N* matrix (*M_ij_*) of average nonbonded interactions between pairs of residues is built first. This energy matrix is then simplified through eigenvalue decomposition. Analysis of the *N* components of the eigenvector associated with the lowest eigenvalue was shown to identify strong interaction centers. This map of pair interactions is subsequently analyzed in light of the topological information summarized by the contact matrix associated to a certain structure. The resulting filtered matrix can be used to identify local couplings characterized by energetic interactions of minimal intensities. In fact, while local low-energy couplings identify those sites in which interaction-networks are not energetically optimized, low-intensity couplings between distant residues in the structure are only a trivial consequence of the distance-dependence of energy functions.

Local low-energy coupled regions can thus be considered as the “soft spots” needed to interact with potential binding partners (in contrast with the “hot spots” characterized by high coupling intensities). Given the low intensity constraints to the rest of the structure, these sub-structures would be characterized by dynamic properties that allow them to visit multiple conformations, a subset of which can be recognized by the antibody to form a complex [46,47].

**Figure 5:**
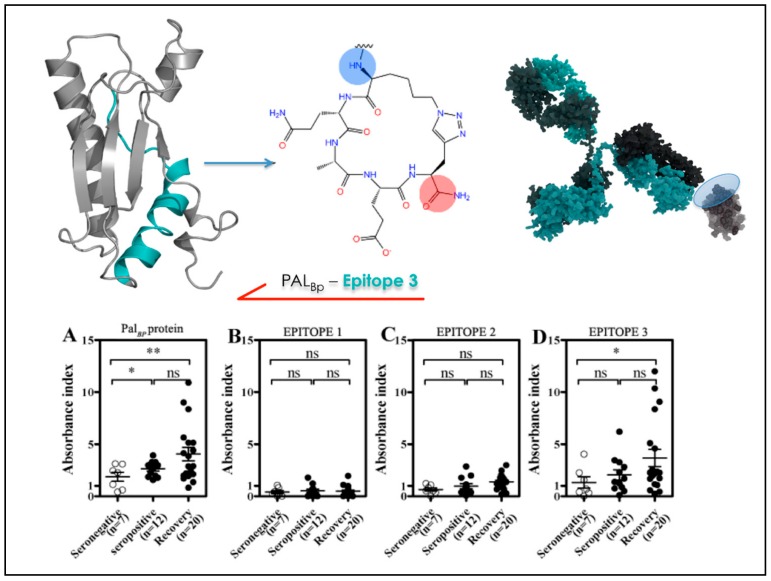
Identification, chemical modification and immunodiagnostic test of the epitope sequence derived from BPSL2765 (PAL_Bp_).

After validating the predictions against the crystal structures of known Ab-Ag complexes, we set out to apply the matrix of local coupling energies (MLCE) approach in a predictive and design-oriented fashion. The first instance in which the method was applied focused on the discovery and design of reactive epitopes from the antigens of the bacterium *Burkholderia pseudomallei* (Bp), the etiological agent of melioidosis. The latter is a severe respiratory infection against which no rapid and efficient diagnostic method or vaccination strategy exists. Several immunoreactive proteins were identified through an RV strategy. The crystal structure of one of these antigens, OppA (Bp), was solved at 2.1 Å resolution and was the basis for MLCE analysis that returned three potential epitopes (Figure 5). Once identified, mimics of the potential epitopes were synthesized in peptidic form and successively tested for their immunoreactivity against sera from healthy seronegative, healthy seropositive, and recovered melioidosis patients. The synthetic peptides allowed the different patient groups to be distinguished, underlining the potential of this approach. These results were a first remarkable illustration of the feasibility of a structure-based epitope discovery process, whose application could effectively expand the understanding of the physico-chemical determinants of protein-protein interactions to the development of designed diagnostic molecules [56].

**Figure 6:**
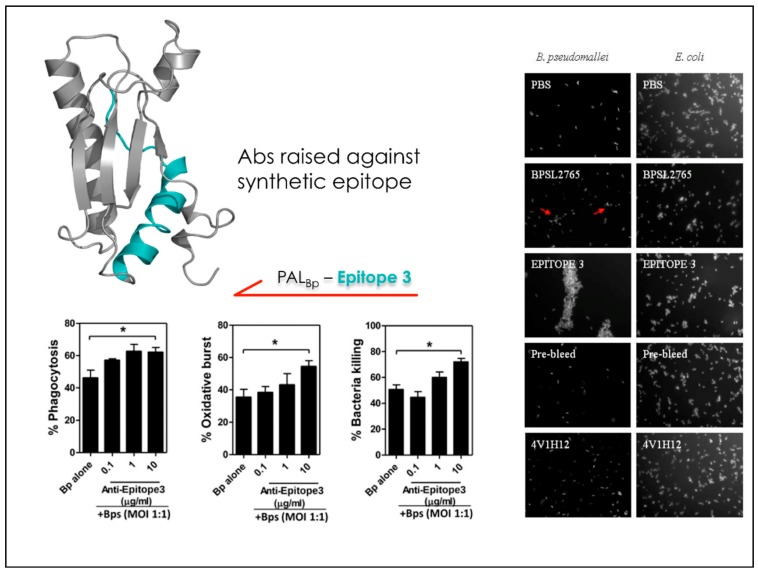
The epitope sequence derived from BPSL2765 (PAL_Bp_) is able to elicit bactericidal antibodies.

Starting from the resolution of the structure of a second *Burkholderia* antigen, namely BPSL2765, the approach was extended to the production of bactericidal antibodies. Based on the structure, MLCE, coupled to in vitro mass-spectrometry mapping, identified a sequence within the antigen that, when engineered as a synthetic peptide, was selectively immunorecognized to the same extent as the recombinant protein in sera from melioidosis patients. Next, the peptide was employed to elicit Abs that were subsequently tested in bacterial killing experiments and antibody-dependent agglutination tests. Importantly, the Abs produced against the designed synthetic peptide turned out to induce the killing of *B. pseudomallei* at levels higher than the Abs raised against the full length protein [57] (Figure 5 and Figure 6). In this case, our strategy represented not only a step in the development of immunodiagnostics, but also a first step in the engineering of antigens and production of specific antibodies for vaccine development.

MLCE was further applied to proteins constituent of the flagella of the bacterium. Flagella are used by the bacterium to move in the environment and are conceivably the first parts of the pathogen that come into contact with the host. MLCE epitope prediction was applied to *B. pseudomallei* flagellar hook-associated protein (FlgK(Bp)) [58,59], allowing us to predict three antigenic regions that locate to discrete protein domains and may work as vaccine components. Another component of the flagella is the large protein flagellin (FliC(Bp)). Interestingly, in this case, three predicted epitopes, when synthesized and tested as free peptides, turned out to be both B and T cell FliC(Bp) epitopes: they were immunoreactive against human IgG antibodies and elicited cytokine production from human peripheral blood mononuclear cells. Furthermore, two of the peptides (F51-69 and F270-288) were found to be immunodominant, with their antibodies enhancing the bactericidal activities of purified human neutrophils [60]. Together with the previously reported ones, these epitopes may represent potential melioidosis vaccine components.

In general, it is tempting to suggest that the possibility to predict the parts of a protein (antigens) endowed with antibody recognition/binding properties and the demonstration of their reactivity in the form of isolated peptides can open up new venues for diagnosis and treatment. In the case of diagnostics, for instance, multiple predicted binding sequences can be displayed on microarrays for medium-high throughput analysis of their interaction profiles: in a notable instance, predicted peptides were optimized for oriented display on microarray plates and proved to be efficient in the rapid diagnosis of *Burkholderia* infections in cystic fibrosis (CF) patients [61]. To mimic conformational epitopes, oriented and spatially controlled co-immobilization of predicted epitope sequences that are spatially proximal in the Zika virus NS1 protein, showed the ability to cooperatively interact to provide enhanced immunoreactivity with respect to single linear epitopes [62].

## 4. Conclusions and Perspectives

The data described above indicate that it is becoming possible to apply rational methods to target difficult protein-protein interactions, both through small molecules and through the harnessing of the reactivity towards large biological molecules as antibodies. We suggest that these methods of drug and peptide design could be conceivably coupled to the design of polyvalent systems that allow the simultaneous binding of multiple ligands to a certain target, mimicking the types of interactions that are widespread in biology [63]. The availability of chemical synthesis methods for the access to complex mimics of natural products or chemical-biology probes [64,65,66,67,68], and the explosion of chemical methods for the display of multiple ligands (through nanoparticles, bio-inspired polymers etc...) can indeed help the development of multivalent systems that we see as potentially suitable for vaccination and patient diagnostics: in these cases, the simultaneous presentation of multiple determinants of Ab-recognition from the antigens of a certain pathogen may help trigger protective response against it [69,70,71,72]. In the case of small molecule drugs, multi-presentation approaches may become particularly useful when targeting large multi-component complexes. In our view, computational chemistry approaches are set to become in the next few years more and more instrumental and integrated with chemical biology and drug design approaches, increasing our understanding of how biological systems work and translating this knowledge into new molecules with interesting therapeutic potential.
